# Etiology of acute otitis media and phenotypic-molecular characterization of *Streptococcus pneumoniae* isolated from children in Liuzhou, China

**DOI:** 10.1186/s12879-019-3795-8

**Published:** 2019-02-15

**Authors:** Jinjian Fu, Ling Li, Zhuoxin Liang, Shaolin Xu, Nan Lin, Peixu Qin, Xiaohua Ye, Eric McGrath

**Affiliations:** 1 Department of Laboratory, Liuzhou Maternity and Child Healthcare Hospital, Liuzhou, China; 2 School of Public Health, Guangdong Pharmaceutical University, 283# Jianghai Dadao, Haizhu District, Guangzhou, 510310 China; 3 Department of Pediatric, Liuzhou Maternity and Child Health Care Hospital, Liuzhou, Guangxi China; 4 Department of Otolaryngology, Liuzhou Maternity and Child Health Care Hospital, Liuzhou, Guangxi China; 5 Children’s Hospital of Michigan, Detroit, MI USA; 6 Wayne State University School of Medicine, Detroit, MI USA

**Keywords:** Acute otitis media, *Streptococcus pneumoniae*, Children, Vaccine, Molecular characteristics

## Abstract

**Background:**

The etiology and epidemiology of acute otitis media (AOM) are poorly understood in China. This study aimed to describe the etiology of AOM and the phenotypic and molecular characteristics of AOM-causing *Streptococcus pneumoniae* (*S.pneumoniae*) recovered from Chinese children.

**Methods:**

A retrospective study was conducted to enrol patients younger than 18 years diagnosed as AOM. Middle ear fluid specimens were collected then cultured for bacterial pathogens. All *S.pneumoniae* isolates were tested for antibiotic susceptibility, serotypes, virulence genes, antibiotic resistant determinants and sequence types.

**Results:**

The dominant otopathogen among AOM children was *S.pneumoniae* (54.4%). Among *S.pneumoniae* isolates, there were 97.3, 97.3 and 75.7% isolates resistant to erythromycin, tetracycline and trimethoprim-sulfamethoxazole, respectively. There was 72.8% *S.pneumoniae* with multidrug resistance. The dominant sequence types (STs) were ST271 and ST320, whereas the prevailing serotypes were 19F and 19A. The 7-valent and 13-valent pneumococcal conjugate vaccine (PCV) coverage among AOM children were 73.0 and 94.6%, respectively. Additionally, we found that CC271 expressed more of *mef*(A/E) (*P* < 0.001), *pspA* (*P* = 0.022) and *sipA* (*P* < 0.001) than non-CC271 isolates.

**Conclusion:**

The high prevalence of international multidrug-resistant clone (Taiwan^19F^-14) in China necessitates continued dedication to expand PCV13 immunization and better control of antibiotic use in China.

## Background

Acute otitis media (AOM) is one of the most frequently encountered bacterial infections and the leading cause of medical visits and antibiotic prescription in children worldwide [1, 2]. Among the pathogens involved in this infection, *Streptococcus pneumoniae* (*S.pneumoniae*) and *Haemophilus influenzae* (*H.influenzae*) are the principal bacterial otopathogens that account for up to 60–80% of microbiologically confirmed AOM [3, 4]. Concerns have been rising about the major role of *S.pneumoniae* in AOM etiology, which coincides with growing antibiotic resistance rates among *S.pneumoniae* isolates. In China, the rate of penicillin-nonsusceptible *S.pneumoniae* (PNSP) ranges from 11.5–39.4% [5, 6]. The Asian Network for Surveillance of Resistant Pathogens (ANSORP) reported that, in China, the rate of resistance to erythromycin was as high as 96.4%, whilst multidrug resistance was 83.3%, which rank China highest among the 11 enrolled Asian countries surveyed [7].

Increasing rates of resistance of *S.pneumoniae* to commonly used antibiotics highlights the increased urgency for use of vaccines for controlling pneumococcal diseases. The heptavalent pneumococcal polysaccharide conjugate vaccine (PCV7) has had a dramatic effect on reducing the disease burden of pneumococcal diseases globally, including AOM [8, 9]. The increased prevalence of pneumococcal diseases caused by non-vaccine serotypes, especially serotype 19A from an emerging clonal complex (CC) 320, has been well documented in the post PCV7 era [10, 11]. PCV7 has been available as a self-paid vaccine in mainland China since August 2008, but immunization rates were less than 10% in published studies [12, 13].

Despite a well-developed knowledge base of *S.pneumoniae* worldwide, in China, little has been reported on the microbial etiology of AOM and phenotypic-molecular characteristics of *S.pneumoniae,* the predominant otopathogen. Therefore, we conducted this study in China to investigate the bacterial etiology and characterize pneumococcal isolates obtained from children with AOM. Phenotypic and genotypic characteristics of the pneumococcal isolates from AOM patients, including antimicrobial susceptibility, serotypes, multilocus sequence typing (MLST) profiles, and virulence genes of *S. pneumoniae* were also investigated.

## Methods

### Patients and isolates

This study was approved by the Ethics Committee of Liuzhou Maternity and Child Healthcare Hospital, and was performed in accordance with the approved guidelines. Before enrolment of a child into this study, written informed consent was obtained from parents or legal guardians on behalf of the children involved in the study for collection of information and samples.

This retrospective study was conducted from January 1st 2016 to June 30th 2016 in the otolaryngology clinic of Liuzhou Maternity and Child Health Care Hospital. Children aged younger than 18 years old, with no prior AOM at the time of enrolment, were enrolled in this study. AOM was diagnosed by the otolaryngologist or pediatrician after otoscopic examination of the ear drum, confirmation of acute onset of symptoms lasting ≤7 days consistent with AOM and accompanied by one or more of the following symptoms: substantial bulging of the tympanic membrane, marked redness of the tympanic membrane, and a cloudy or purulent effusion was observed.

Middle ear fluid specimens were obtained by either tympanocentesis or collection of pus from draining ears and were immediately delivered to the clinical microbiology laboratory for culture. The specimens were plated on Colombia blood agar with 5% sheep blood and Chocolate agar and placed in 35 °C, 5% CO_2_ incubated for 24 h to 48 h. All agars were purchased from Autobio (Henan, China). *S.pneumoniae* was identified by typical alpha haemolysis and draftsman colonies on the blood agar plate together with optochin test and bile solubility test. *H.influenzae* was identified by growth requirements for factors V and X. The disk of optochin, X, V and X + V were purchased from OXOID (Hampshire, England). The suspected isolates were confirmed by the VITEK 2 Gram Positive Card (*Streptococcus pneumoniae*, *Staphylococcus aureus*, *Streptococcus pyogenes*), Gram Negative Card (*Pseudomonas aeruginosa*), and Neisseria-Haemophilus Card (*Haemophilus influenzae*) using VITEK 2 compact automatic microbial analysis system (Biomérieux, Marcyl’ Etoile, France). Chromogenic Agar medium was used for Candida species identification (Biomérieux) and API 20C AuX (Biomérieux) was used for confirmation. In this study, we only focused on the epidemiology and phenotypic-molecular characterization of *S. pneumoniae*. The pneumococcal isolates were stored at − 80 °C in 10% glycerol-brain heart infusion broth with 50% sheep blood.

### Antibiotic susceptibility test

The antibiotic susceptibility of *S.pneumoniae* isolates were determined by the minimum inhibitory concentration with the VITEK 2 Gram Positive Susceptibility Card-AST-GP68 using VITEK2 systems. Definitions of antibiotic susceptibility were based on the Clinical and Laboratory Antimicrobial Standards Institute (CLSI) 2016 criteria [14]. Current CLSI susceptible, intermediate, resistant (respectively) breakpoints for parenteral penicillin (2 μg/ml, 4 μg/ml, 8 μg/ml; nonmeningitis), oral penicillin (0.06 μg/ml, 1 μg/ml, 2 μg/ml), cefotaxime (1 μg/ml, 2 μg/ml, 4 μg/ml; nonmeningitis), erythromycin (0.25 μg/ml, 0.5 μg/ml, 1 μg/ml), and levofloxacin (2 μg/ml, 4 μg/ml, 8 μg/ml), tetracycline (1 μg/ml, 2 μg/ml, 4 μg/ml), trimethoprim -sulfamethoxazole (0.5/0.95 μg/ml, 2/38 μg/ml, 4/76 μg/ml) were considered. The CLSI QA and QC guidelines were followed for antibiotic susceptibility testing together with *S. pneumoniae* ATCC49619 as the quality control strain. Isolates non-susceptible to at least 3 antibiotic classes were defined as multidrug-resistant *S. pneumoniae.*

### DNA extraction

Chromosomal DNA was extracted from the overnight subculture of *S.pneumoniae* isolates by using Biospin Bacteria Genomic DNA Extraction Kit (Bioer Ltd., Hangzhou, China) according to the manufacturer’s instructions.

### Serotyping

Pneumococcal serotyping was performed using multiplex polymerase chain reaction (m-PCR) methods as described in a previous study [15]. Thirty-six different serotypes were determined by 8 sequential m-PCR reactions and the method used to distinguish serotypes 6A/6B was as reported previously [6].

### Detection of antibiotic-resistant genes

The macrolide-resistant genes *erm*(A), *erm*(B) and *mef*(A/E) were amplified by PCR methods for all erythromycin-resistant isolates [16]. The *tet*(K), *tet*(L) and *tet*(O) genes were amplified by PCR for all tetracycline-resistant isolates using the primers and PCR conditions as previously described [17]. All the PCR products were visualized by 1.5% agarose gel electrophoresis and gold-view staining.

### Detection of virulence genes

Detection of virulence genes (*ply*, *pasA*, *lytA*, *pspA*) and pili genes (*rlrA* for PI-1and *sipA* for PI-2) of pneumococcal isolates were amplified using PCR and all primers, PCR reactions and conditions used were as previously described [17]. The 100 bp plus DNA ladder marker (Takara, China) was used for molecular weight reference.

### Multilocus sequence typing

Sequence types (STs) of pneumococcal isolates were determined using the multilocus sequence typing (MLST) technique. Internal fragments of 7 housekeeping genes (*aroE*, *gdh*, *gki*, *recP*, *spi*, *xpt*, *ddl*) were amplified from chromosomal DNA by PCR [18]. Allelic profiles and STs were assigned by comparison with pneumococcal MLST database (https://pubmlst.org/spneumoniae), and clonal complexes (CCs) were determined using the eBURST algorithm. The definition of a clonal complex is a group in which every strain shares at least five identical alleles out of seven with at least one other genotype in the group [19].

### Statistical analysis

Data were analyzed using descriptive statistics and Pearson’s chi-squared (χ^2^) test or Fisher exact test. A two-sided *P*-value of less than 0.05 was considered as being of statistical significance. All statistical analyses were conducted using SPSS version 20.0 (SPSS Inc. Chicago, Il, USA). PHYLOViZ method (http://www.phyloviz.net) was used to demonstrate the relationship of serotypes and MLST.

## Results

### Microbiology of AOM

Seventy-nine patients were enrolled during the study period. Eighty-four samples were collected from 79 patients, 68 samples from 68 patients were culture positive. The predominant pathogen was *S.pneumoniae* (37 isolates), followed by *H.influenzae* (12 isolates), *Staphylococcus aureus* (7 isolates), *Pseudomonas aeruginosa* (5 isolates), *Streptococcus pyogenes* (4 isolates) and *Candida albicans* (3 isolates). The demographic information of children with AOM is shown in Table 1. Seventy-four of the 79 children with AOM (93.7%) were less than 2 years old, and the majority of the patients were male (58.2%). All 37 children experiencing pneumococcal AOM were less than 2 years old, whilst 62.2% were male.

**Table 1:** The demographic information of children with AOM

Characteristic	AOM		*S. pneumoniae* positive	
	*n*	%	*n*	%
Gender				
Male	46	58.2	23	62.2
Female	33	41.8	14	37.8
Age (months)				
11	20	25.3	12	32.4
12–23	27	34.2	11	29.7
24–59	27	34.2	14	37.8
≥ 60	5	6.3	0	0.0

*AOM*, acute otitis media

### Antibiotic susceptibility of *S. pneumoniae*

In pneumococcal isolates, no isolate was resistant to parenteral penicillin, but the intermediate and resistant rates reached 59.5% (22/37) and 27.0% (10/37), respectively, based on the oral breakpoints. The non-susceptibility rates were low for levofloxacin (2.7%) and chloramphenicol (8.1%), but high for erythromycin (100.0%), tetracycline (97.3%) and trimethoprim-sulfamethoxazole (83.8%) (Table 2). The multidrug-resistant rate of pneumococcal isolates was 72.8%. The most common multidrug-resistant patterns were non-susceptibility to erythromycin/tetracycline/trimethoprim-sulfamethoxazole (59.5%), erythromycin/tetracycline/trimethoprim-sulfamethoxazole/chloramphenicol (8.1%) and erythromycin/tetracycline/trimethoprim-sulfamethoxazole/cefotaxime (5.4%).

**Table 2:** Antimicrobial susceptibility of *S. pneumoniae* isolated from children with AOM

Antibiotic	Susceptible n (%)	Intermediate n (%)	Resistant n(%)	MIC_50_ (μg/mL)	MIC_90_ (μg/mL)
Penicillin Parenteral^a^	36 (97.3)	1 (2.7)	0 (0.0)	0.15	1
Penicillin Oral	5 (13.5)	22 (59.5)	10 (27.0)	0.15	1
Vancomycin	37 (100.0)	0 (0.0)	0 (0.0)	0.5	0.5
Erythromycin	0 (0.0)	1 (2.7)	36 (97.3)	1	2
Levofloxacin	36 (97.3)	1 (2.7)	0 (0.0)	0.5	1
Tetracycline	1 (2.7)	0 (0.0)	36 (97.3)	4	16
Trimethoprim-sulfamethoxazole	6 (16.2)	3 (8.1)	28 (75.7)	0.5/0.95	0.5/0.95
Linezolid	37 (100.0)	0 (0.0)	0 (0.0)	0.5	0.5
Chloramphenicol	34 (91.9)	0 (0.0)	3 (8.1)	2	4
Cefotaxime^a^	31 (83.8)	4 (10.8)	2 (5.4)	1	2

*AOM*, acute otitis media

^a^2016 CLSI breakpoints were considered for nonmeningitis

### Serotyping, vaccine coverage and sequence types of *S. pneumoniae*

**Fig. 1:**
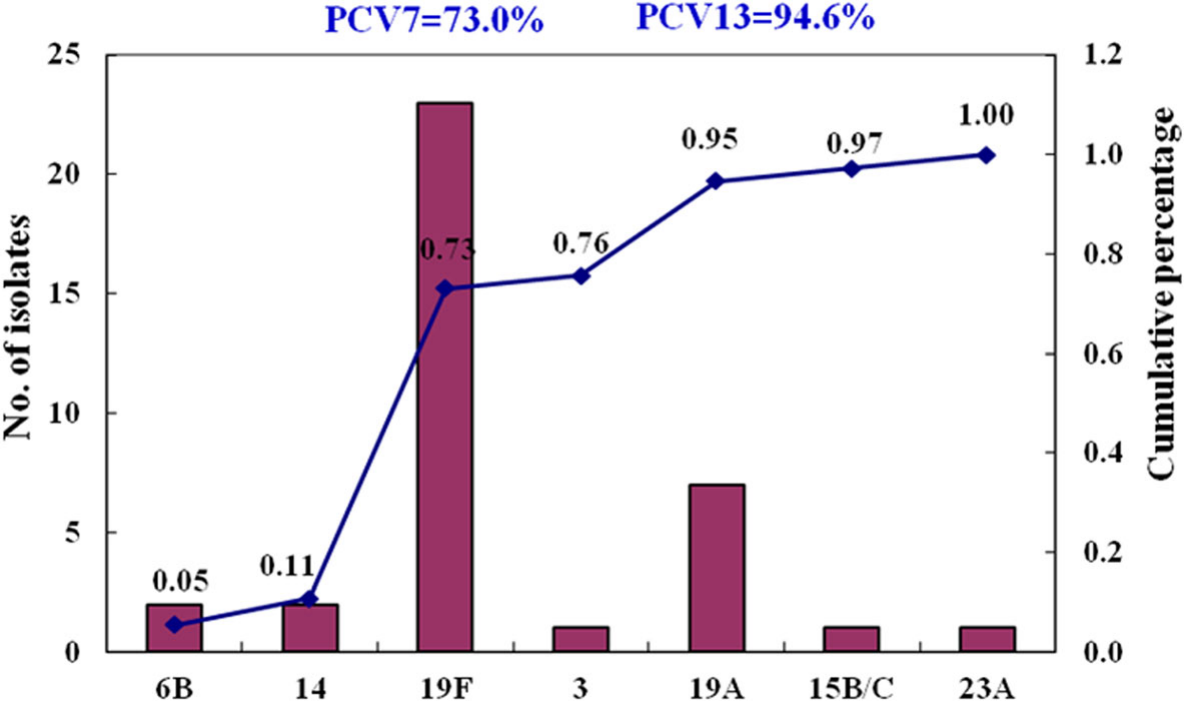
Serotype distribution in 37 *S.pneumoniae* isolates recovered from AOM patients (PCV7 includes serotypes 4, 6B, 9 V, 14, 18C, 19F, and 23F; PCV13 includes serotypes 1, 3, 4, 5, 6A, 6B, 7F, 9 V, 14, 18C, 19A, 19F, and 23F)

**Fig. 2:**
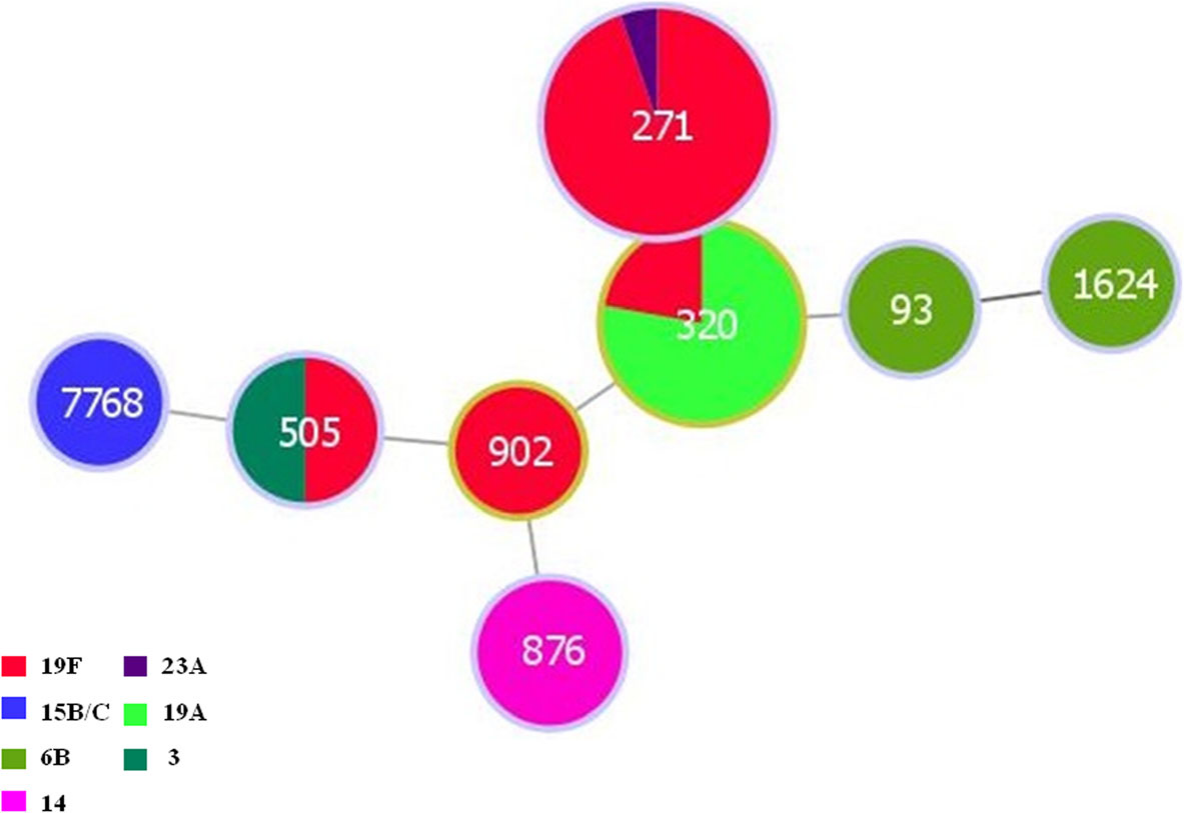
The relationship between serotype and genotype of pneumococci recovered from AOM patients

Among the 37 *S.pneumoniae* isolates, the predominant serotypes were 19F (62.2%) and 19A (18.9%), accounting for 81.1% of all the isolates. The pneumococcal vaccine coverage rate for the pneumococcal isolates was 73.0% for PCV7, but high at 94.6% for PCV13 (Fig. 1). All 36 *S.pneumoniae* isolates were successfully typed by MLST, only one isolate, serotype 19F, failed to sequence. Among the 8 STs identified, the most common STs were ST271 (*n* = 19, 51.4%) and ST320 (*n* = 9, 24.3%), with the majority of ST271 and ST320 isolates serotyped as 19F and 19A, respectively (Fig. 2). The eBURST analysis revealed 1 CC and 6 singletons containing 75.7 and 21.6% of the isolates, respectively, with the predominant CC271 including 28 isolates (75.7%).

### Antibiotic resistance genes and virulence genes of *S. pneumoniae*

Among the 37 pneumococcal isolates (Table 3), 94.6% (35 isolates) carried *erm*(B*)* gene, 64.9% (24 isolates) carried *mef*(A/E) gene, 94.6% (35 isolates) carried *tet*(M) gene, and only 1 isolate carried *tet*(O) gene. Notably, about 94.6% of *S.pneumoniae* isolates were co-resistant to tetracycline and erythromycin, carrying both *erm*(B) and *tet*(M) genes, whilst none of the 37 isolates contained macrolide or tetracycline resistance genes, such as *erm*(A), *tet*(K) or *tet*(L). Tetracycline phenotypic resistance results showed that over 91.9% (34/37) of isolates carried *tet*(M) gene. In comparison, the macrolide phenotypic resistance results, showed 94.6% (35/37) of isolates carried *erm*(B*)* gene, while only 62.2% (23/37) carried *mef*(A/E) gene, indicating good correlation between phenotypic-genotypic results in terms of tetracycline and erythromycin resistance. In all of the isolates, 97.3% (36 isolates) carried both pneumococcal virulence genes *lytA* and *ply*, and 83.8% (31 isolates) carried *pspA* gene. The *rlrA* gene was identified in 22 of 37 (59.5%) isolates, and *sipA* was detected in 67.6% (25 isolates) of the pneumococcal isolates.

**Table 3:** Molecular characteristics of *S. pneumoniae* isolated from children with AOM

ST,serotype (*n*)	Resistance genes n(%)			Virulence genes n(%)				
	*erm*(B)	*mef*(A/E)	*tet*(M)	*ply*	*lytA*	*pspA*	*rlrA*	*sipA*
ST271,19F(18)	17 (45.9)	15 (40.5)	18 (48.6)	18 (48.6)	18 (48.6)	17 (45.9)	13 (35.1)	17 (45.9)
ST271,23A(1)	1 (2.7)	1 (2.7)	1 (2.7)	1 (2.7)	1 (2.7)	1 (2.7)	0 (0.0)	0 (0.0)
ST320,19F(2)	1 (2.7)	1 (2.7)	1 (2.7)	2 (5.4)	2 (5.4)	2 (5.4)	2 (5.4)	2 (5.4)
ST320,19A(7)	7 (18.9)	5 (13.5)	7 (18.9)	7 (18.9)	7 (18.9)	6 (16.2)	4 (10.8)	5 (13.5)
ST505,3 (1)	1 (2.7)	0 (0.0)	1 (2.7)	1 (2.7)	1 (2.7)	0 (0.0)	0 (0.0)	0 (0.0)
ST505,19F(1)	1 (2.7)	0 (0.0)	1 (2.7)	1 (2.7)	1 (2.7)	0 (0.0)	0 (0.0)	0 (0.0)
ST876,14 (2)	2 (5.4)	0 (0.0)	2 (5.4)	2 (5.4)	2 (5.4)	2 (5.4)	0 (0.0)	0 (0.0)
ST902,19F(1)	1 (2.7)	1 (2.7)	1 (2.7)	1 (2.7)	1 (2.7)	0 (0.0)	1 (2.7)	1 (2.7)
ST1624,6B(1)	1 (2.7)	0 (0.0)	1 (2.7)	1 (2.7)	1 (2.7)	1 (2.7)	1 (2.7)	0 (0.0)
ST7768,15B/C(1)	1 (2.7)	0 (0.0)	1 (2.7)	1 (2.7)	1 (2.7)	1 (2.7)	1 (2.7)	0 (0.0)
ST93,6B(1)	1 (2.7)	0 (0.0)	1 (2.7)	1 (2.7)	1 (2.7)	1 (2.7)	0 (0.0)	0 (0.0)
Untypeable,19F(1)	1 (2.7)	0 (0.0)	0 (0.0)	0 (0.0)	0 (0.0)	0 (0.0)	0 (0.0)	0 (0.0)
Total	35 (94.6)	23 (62.2)	35 (94.6)	36 (97.3)	36 (97.3)	31 (83.8)	22 (59.5)	25 (67.6)

NOTE. Values are number of isolates

*AOM*, acute otitis media

### Relations between ST types, serotypes, and resistance-virulence characteristics

All the serotype 19A isolates belonged to ST320 (Table 3). Overall, 73.3% of serogroup 19 harbored *me*f(A/E), while 14.3% of the non-serotype 19 harbored *mef*(A/E) (*P* = 0.007). The same difference was observed in *sipA* (*P <* 0.001). Up to 80% (22 isolates) of CC271 clones expressed *mef*(A/E), and only 11.1% of other isolates expressed *mef*(A/E), indicating significant difference between two groups (*P <* 0.001). Similar significant differences were observed in the carriage of *pspA* (*P* = 0.022) and *sipA* (*P* < 0.001) genes expressed in CC271 clones and other isolates. As to antibiotic resistance genes and pneumococcal virulence genes, we observed that ST271 expressed more of *mef*(A/E) (*P =* 0.004) and *sipA* (*P =* 0.001) than non-ST271 isolates.

## Discussion

AOM is a common upper respiratory tract infection among children younger than 5 years old. The monitoring of etiology of AOM, antibiotic resistance and the molecular background of AOM-causing *S.pneumoniae* provides opportunities to identify changes in the predominant bacteria and the impact of changing antibiotic resistance and prevalence of virulence genes within these bacteria that may result from environmental selection pressures such as pneumococcal immunization and antibiotic use. In this retrospective study, we found that *S.pneumoniae* was the most prevalent pathogen causing for AOM in the recruited children and was isolated from 46.8% of the cohort. The high proportion of the pneumococcal isolates resistant to erythromycin, tetracycline and trimethoprim-sulfamethoxazole (59.5%) is consistent with the Suzhou study of nasopharyngral isolates [20]. Interestingly, the proportion of pneumococcal isolates that were resistant to penicillin, was inconsistent with previous reports from China that demonstrated a high prevalence of resistance to penicillin [6, 20]. This inconsistency may be explained by the prescribing pattern in Liuzhou, which uses macrolide antibiotics such as erythromycin and azithromycin, not penicillin, as the first option prescription for pediatric patients. The second reason may be that the isolates we collected were from middle ear fluid of pediatric patients with AOM, which were reported more frequently in the community. There was report that the *S.pneumoniae* isolates were more susceptible to penicillin within the community than in the medical places [21], which may explain the inconsistency of our results from other reports collected isolates from hospitalized children with a high prevalence of resistance to penicillin [6].

It was reported that the resistance of *S.pneumoniae* to the third-generation cephalosporin was increasing in some area of China. For example, in Suzhou, among the *S.pneumoniae* isolates collected from the children with AOM, the penicillin resistant *S.pneumoniae* (PRSP) was 10.2%, while the resistance rate of cefotaxime (CTX) was 34.3% [20]. Among the 58 *S.pneumoniae* CC271 clones of the serotype 19F collected from children with non-meningitis in Beijing, the non-susceptible rate to ceftriaxone was 96.4%, while only 8.6% isolates was penicillin non-susceptible *S.pneumoniae* (PNSP) [22]. In our study, only 1 of the *S.pneumoniae* isolates was determined as PNSP, but as many as 16.2% (6/37) of isolates were determined as CTX non-susceptible *S.pneumoniae* (using non-meningitis breakpoints based on the CLSI criteria [14]), which was consistent with the previous reports [5, 6, 20, 22]. Interestingly, all 7 of these isolates are serotype 19F, ST271clone. These findings are supported by a report from Ip M et al. [23] which observed that in Hongkong 2011, the rates of PNSP and CTX non-susceptibility were 23.3 and 30.3% respectively but that all of the CTX resistant isolates were attributed to the single serotype 19F, CC271, with only 25.3% of these isolates was penicillin resistant [23]. Resistance of *S.pneumoniae* to the third-generation cephalosporin CTX was increasing in the above areas due to the acquisition of an additional 19 amino acid substitutions in penicillin binding protein 2b (PBP2b) and PBP2x through genome recombination events [23]. Furthermore, the pbp2b gene of *S.pneumoniae* has very low affinity for cephalosporins and inactivation of this gene seems not to be working on the CTX [24] thus cause the high rate of non-susceptible CTX but low rate of PNSP in our study.

Macrolide resistance rates of pneumococcal isolates vary greatly throughout the world however the pneumococci resistance rate to erythromycin was 72.7% in Asia, with the highest rate in China (96.4%) as reported by ANSORP [25]. It is noteworthy that pneumococci harboring both *erm*(B) and *mef*(E) genes continue to be reported, with a global prevalence of 16.4% among macrolide-resistant pneumococcal isolates [26]. Overall in this study, 94.6 and 62% of the macrolide-resistant pneumococci were positive for *erm*(B) and *mef*(A/E), respectively. The present study also showed that 78.6% of the Taiwan^19F^-14 clone harbored dual positive genes, while only 11.1% of other STs harbored both genes which is consistent with a previous report that *erm*(B) and *mef* genes have been related to the Taiwan^19F^-14 clone [265]. As a result of the insertion of *erm*(B) into conjugative transposons of the Tn916 family, most of pneumococcal isolates carried *erm*(B) genes are also resistant to tetracycline, typically carrying *tet*(M) [22]. In our study, most of Taiwan^19F^-14 isolates harbored *erm*(B), *mef*(A/E) and *tet*(M) genes, while only 41.2% of other isolates expressed these three genes. These findings suggest that the widespread of highly resistant CC271 maybe driven by the selective pressure of the antibiotic use, which poses a serious public health concern for increasing treatment failure with the empirical usage of macrolide antibiotics.

Two serotypes (19F and 19A) were the most prevalent serotypes (81.1% of these pneumococcal isolates) in our study, which was consistent with a report from children with AOM from Suzhou (80.5%) [20]. Reports for pneumococcal isolates identified from other specimens such as respiratory and nasopharynx revealed that 19F, 14, 23F, 6B and 19A were the most common serotypes [6]. In our study, the serotype coverage rate of PCV7 and PCV13 was 73.0 and 94.6%, respectively. Together with the reports from Suzhou and Shenzhen, China [5, 6], these results support that the increasing coverage of PCV13 was mainly because of the high occurrence of serotype 19A and suggest that expanded use of PCV13 in China, may significantly reduce the prevalence of pneumococcal diseases (especially the invasive pneumococcal diseases) resulting from high prevalence of serotype 19A, although serotype replacement always remains a concern.

Using the eBURST analysis both ST271 and ST320 are members of CC271 which accounts for 75.7% of the pneumococcal isolates in this study. CC271 is a multidrug resistant pneumococcal clone (Taiwan^19F^-14) which has been described internationally [27]. The clone described in this study is classified as multi drug resistant as it is resistant to penicillin, erythromycin and tetracycline which also carry the Tn2010 genetic element encoded on the *erm*(B), *mef*(E) and *tet*(M) genes [28]. Although not examined in the present study, previous studies have shown the increase of CC271 from 14.3% in 1997 in Beijing, China which rose to 92.0% in 2010 [29]. CC271 has also previously been described as the most prevalent clone causing invasive pneumococcal disease [30]. This clone appears to have become successful due to a number of factors, for example increasing international movements, selective pressure due to antibiotic use [29], this clone is now distributed globally and is widespread having been described in Colombia, Spain, USA and China [31–34]. Increased resistance to antibacterial medications continue to pose great challenges for treating the pneumococcal diseases.

Recent evidence has revealed that the pneumococcal serotype 19A/ST320 clone, driven from Taiwan^19F^-14 clone (ST236), switched serotype in response to the selective pressures of the immunization of PCV7 and has become prevalent in many countries, within Asia including China [33, 34]. This clone afflicted Chinese children before the introduction of PCV7 in 2008 [27] and that serotype 19A/ST320 has become resistant to multiple antibiotics [34]. Notably, serotype 19A/ST320 was also found in our study, and most of the serotype 19A/ST320 isolates were multidrug-resistant, and harbored *erm*(B), *mef*(A/E) and *tet*(M) genes. These findings suggest that antibiotic prescribing patterns in China may play a significant role in the switching serotype from 19F to 19A and their homogeneous genetic background. The high prevalence of serotype 19A/ST320 clone suggests that PCV13 should be included into the Chinese Expanded Program on Immunizations, which could further reduce the burden of pneumococcal diseases.

Vaccine candidates such as *pspA*, a protein-based vaccine candidate has been demonstrated to be independent of capsular serotypes [35]. This high identical capacity made *pspA* as a good vaccine candidate to improve the protecting efficacy of the pneumococcal isolates compared with the current usage of serotype-based vaccines, since *pspA* could avoid the serotype replacement which has been observed in the current usage of pneumococcal vaccines [36]. As serogroups 6 and 19 seem to be associated with *pspA* families, this suggests that vaccines covering the *pspA* family might be a better option than the current pneumococcal vaccines which are dependent on the presence of certain capsules. In line with a previous study [35], the current results confirmed that 86.5% of the pneumococci harbored *pspA* gene and was associated with serotypes 6B, 15B/C, 14, 23A, 19A and 19F, further supporting the consideration of *pspA* as a leading candidate for future vaccine development.

Pilus islet has recently been implicated in its virulence for assisting the pneumococci with adherence to host tissue [37] and its capacity of initiating mucosal damage and triggering mucosal inflammation means the pilus islet is able to invade the tissue [37]. A prior study emphasized the high prevalence of the pilus islet among capsular serotypes included in PCV7 such as 19F [38], and the re-emergence of pilus islet among non-vaccine serotype of pneumococcal isolates also has been reported recently [39]. Both the PI-1 (*rlrA*) and PI-2 (*sipA*) genes have previously been associated with the Taiwan^19F^-14 clone (CC271), we describe a genetic relatedness to CC271 together with serotypes 19F and 19A and found the strains also carried the *erm*(B), *mef*(A/E) and *tet*(M) genes. This suggests there may be a clinical relevance between the presence of these genes and AOM in Chinese children. Our work suggests that there is a clonal relationship between the virulence gene, serotype and presence of the pilus islet within CC271 [40].

There were several limitations within the present study. An obvious limitation of the study is the retrospective design and the small sample size, which may limit the generalizability of our study results.. Second, this study mainly focused on children with AOM obtained by tympanocentesis and these isolates may not be representative of the whole spectrum of otitis media, however, the PCR detection of the whole genetic background of pneumococcal isolates including serotype, MLST, antibiotic determinants and virulence factors represent important strengths of the study design. The third one was that we didn’t use controls in the PCRs. However, the 100 bp plus DNA ladder marker was used for molecular weight reference as the references described to make sure we get the right results.

## Conclusions

In conclusion, the emergence of multidrug-resistant *S.pneumoniae* 19A/ST320 harboring multiple antibiotic resistance genes and virulence genes is both concerning and a great challenge to treat the pneumococcal diseases which warrants the continued monitoring of pneumococcal diseases in Chinese children. The high prevalence of international multidrug-resistant clone (Taiwan^19F^-14) in western China necessitates the continued dedication to expand PCV13 immunization in national vaccination programs along with efforts to curb and control the overuse of antibiotics in Chinese children.

## Abbreviations

ANSORP: Asian Network for Surveillance of Resistant Pathogens
AOM: Acute otitis media
CC: Clonal complex
CTX: Cefotaxime
*H.influenzae*: *Haemophilus influenzae*
MLST: Multilocus sequence typing
PCV: Pneumococcal conjugate vaccine
PNSP: Penicillin-nonsusceptible *S.pneumoniae*
PRSP: Penicillin resistant *S.pneumoniae*
*S.pneumoniae*: *Streptococcus pneumoniae*
STs: Sequence types
